# Exploring neurocognitive inefficiencies in anorexia nervosa

**DOI:** 10.1186/2050-2974-3-S1-O67

**Published:** 2015-11-23

**Authors:** Marion Roberts, Kate Tchanturia, Janet Treasure

**Affiliations:** 1 King's College London, Department of Psychological Medicine, Institute of Psychiatry, Psychology and Neuroscience, London, UK; 2 Thrive Eating Disorder Service, Auckland, New Zealand

## 

Neurocognitive findings in the field of eating disorders have consistently highlighted two aspects of executive functioning that pose particular difficulties for those with anorexia nervosa (AN): poor set-shifting and weak coherence. The current piece of research aims to explore the prevalence and clinical correlates of women with AN that show a neurocognitive profile consistent with both poor set-shifting and weak coherence. Fifty-four outpatient women with AN were administered a semi-structured clinical interview and six neurocognitive tasks assessing neurocognitive profile, together with self-report measures. One in five women with current AN met criteria for both poor set-shifting and detail-focussed neurocognitive inefficiencies. Compared to those with one, those with both inefficiencies showed a more severe clinical picture and poorer prognostic factors. Identification of the subgroup of those with AN that present with both neurocognitive inefficiencies simultaneously may flag cases of higher clinical risk where a more targeted intervention may be required. Clinical implications will be discussed, together with an update on intervention research based on neurocognitive profile (cognitive remediation therapy).

